# Topical Treatment With Clindamycin for Confluent and Reticulated Papillomatosis in a Pregnant Woman

**DOI:** 10.7759/cureus.114229

**Published:** 2026-08-09

**Authors:** Caleb M Findley, Buchi Neita, Joseph M Dyer

**Affiliations:** 1 Osteopathic Medicine, Philadelphia College of Osteopathic Medicine, Suwanee, USA; 2 Dermatology, Epiphany Dermatology, Peachtree City, USA; 3 Dermatology, Philadelphia College of Osteopathic Medicine, Suwanee, USA

**Keywords:** carp, confluent and reticulated papillomatosis, pregnancy, pregnant, topical clindamycin

## Abstract

Confluent and reticulated papillomatosis (CARP) is a rare skin condition that typically shows an effective response to oral minocycline. In this case, we offer support for an alternative therapy with topical clindamycin and glycolic acid for seven weeks, leading to full resolution of CARP in a gravida 2 para 1 woman in her first trimester of pregnancy. Limited evidence exists of topical therapies suitable for use in pregnant individuals without underlying contraindications. Pregnancy limits the use of tetracyclines and retinoid therapies. Therefore, topical clindamycin and glycolic acid offer a low-risk treatment, expanding upon the available options for non-systemic therapies in the management of CARP.

## Introduction

Confluent and reticulated papillomatosis (CARP) is an uncommon benign dermatosis characterized by brown hyperkeratotic papules coalescing into reticulated plaques with distribution mainly in the intertriginous areas, trunk, or neck [1]. Due to the low public health burden of this condition and its rarity, formal epidemiological studies at the global and regional level are limited [2]. Most presentations commonly occur in individuals aged 8 to 32 years old, more often during puberty around 15 years of age, with a slight predominance in female patients [2]. While the etiology remains undetermined, some proposed mechanisms include abnormal differentiation of keratinocytes, abnormal host reaction to fungi or bacteria, and abnormal endocrine function associated with obesity or hyperinsulinemia [2].

The gold standard therapy for CARP has been recognized as oral minocycline [1]. Other oral treatments with decreased side effect profiles, like azithromycin, have demonstrated benefit as well [2,3]. Oral antibiotics, however, may not be appropriate choices for those who are pregnant, have allergies, or refuse systemic therapies. Topical therapies like tazarotene gel, selenium sulfide, ketoconazole, or calcipotriol have shown effectiveness to a lesser degree than oral antibiotics and are represented by a small subset of published literature [2,3]. A recent case report documented the clearance of CARP within 3 months using combined topical clindamycin phosphate 1.2%/adapalene 0.15%/benzoyl peroxide 3.1% gel with a sodium hypochlorite body wash [4].

In this case, we report a gravida 2 para 1 woman in the first trimester of pregnancy presenting with CARP. The patient had complete resolution of physical findings following the use of daily topical clindamycin phosphate 1% lotion combined with glycolic acid 5% gel-cream body wash. Given the paucity of documented reports demonstrating the benefits of topical clindamycin in improving CARP, this topical treatment regimen may offer a valuable therapeutic option for patients with a favorable side-effect profile.

## Case presentation

**Figure 1:**
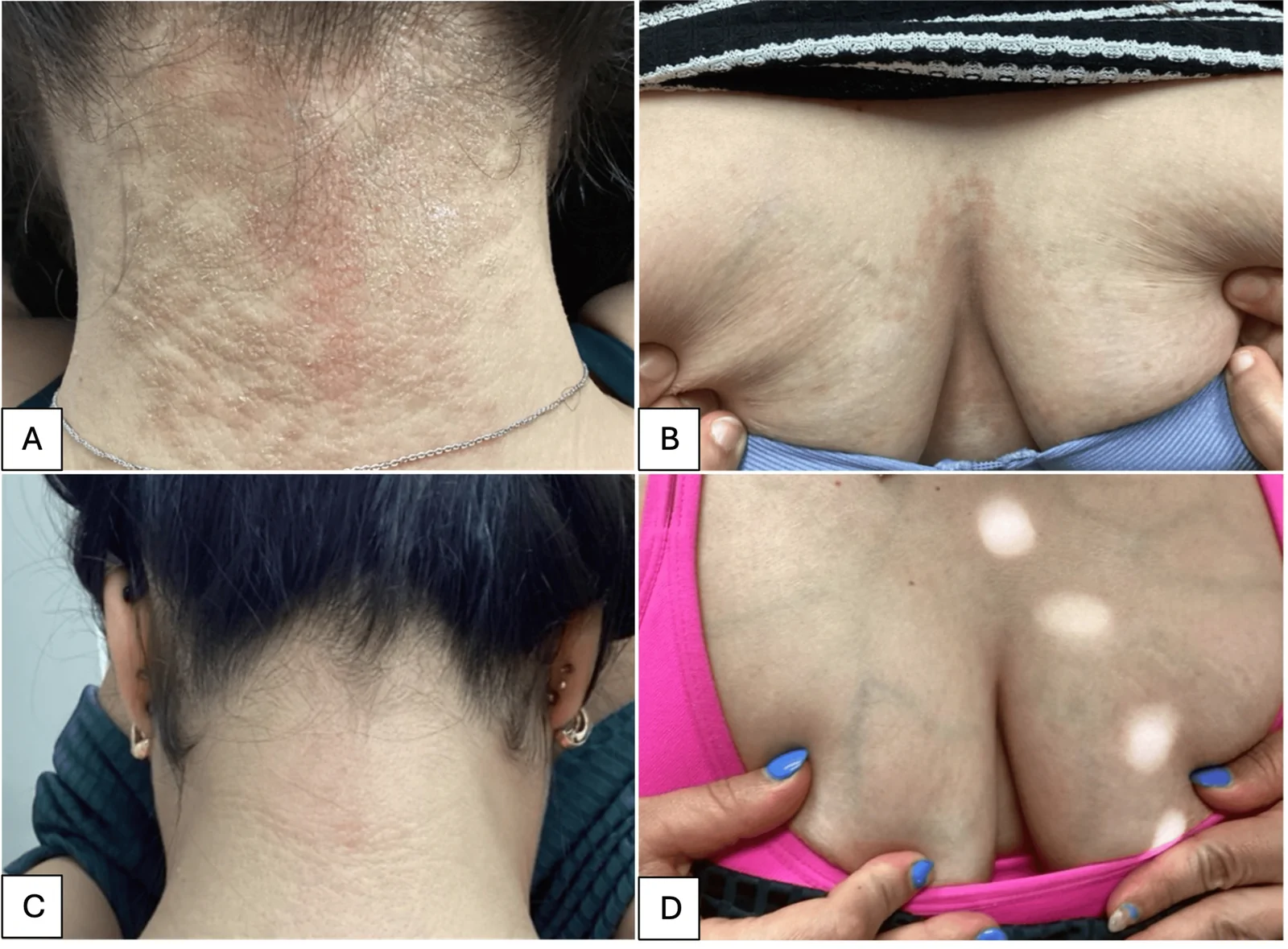
A and B: Reticulated brown plaque with scale on the posterior neck and intermammary cleft; C and D: Resolution of rash after seven weeks of treatment with topical clindamycin 1% and glycolic acid

A 33-year-old Latina woman at 8 weeks' gestation presented with a gradual new-onset darkening of skin on the central chest and posterior neck with concerns of irritation and an 8/10 burning sensation. The patient reported a similar occurrence during her first pregnancy. Aside from seborrheic dermatitis controlled with topical desonide 0.05% and sulconazole 1% cream, as well as Crohn's disease treated with ustekinumab 90 mg/mL subcutaneous injection, her past medical history was otherwise unremarkable. The patient denied alcohol or tobacco usage. Physical examination revealed thin hyperpigmented papules coalescing into reticulated light-brown plaques on the intermammary cleft and posterior neck, with faint hyperkeratotic scale (Figure 1). Differential diagnoses consisted of tinea versicolor or CARP. Initially, a sample was obtained with a 15-blade to scrape the skin from the posterior neck and placed on a glass slide covered with KOH (potassium hydroxide) solution. Findings were within the normal realm and negative for short, wide hyphae or clusters of spores. The patient was treated empirically with sulconazole 1% cream daily for four weeks.

At the follow-up appointment, treatment with antifungal cream yielded no response. The persistence of reticulated scaly hyperpigmented papules and plaques within the same location led to the clinical diagnosis of CARP (Figure 1). Due to pregnancy and patient preference, the traditional choice of oral minocycline or alternative therapies like macrolides and topical retinoids was not selected. Instead, topical clindamycin phosphate 1% lotion twice a day was initiated alongside an over-the-counter glycolic acid 5% gel-cream body wash once daily to address the possible underlying abnormal host reaction to bacteria and keratinocyte differentiation. The patient was instructed to generously apply glycolic acid 5% gel-cream body wash to affected areas in the shower prior to rinsing and applying 1 FTU (fingertip unit) of topical clindamycin phosphate 1% lotion to the intermammary cleft and 1 FTU to the posterior neck on dry skin. At the seven-week follow-up appointment, the patient had complete resolution of symptoms without any residual rash on the chest and posterior neck. Due to the effective response, the patient was instructed to continue use of topical therapy as needed if there was recurrence during the postpartum period.

## Discussion

The diagnostic criteria of CARP have been proposed as the presence of scaly papules and patches or plaques that appear partly reticulated, commonly distributed on the upper trunk and neck [1]. Negative fungal staining and failure of resolution with topical antifungals, with a robust response to oral minocycline, corroborate the diagnosis [1]. These criteria were consistent with the classic morphology and distribution of CARP in this 33-year-old pregnant woman with a hyperpigmented confluent plaque in the center and a reticulated pattern at the periphery of the chest and posterior neck. The subjective findings of burning and irritation were unique to this case, as CARP typically is associated with mild pruritus or an absence of physical discomfort [2]. Moreover, standard treatment with oral minocycline was unable to be given due to teratogenicity.

Oral minocycline is a tetracycline-class antibiotic that is contraindicated in pregnancy due to the risk of reduced bone growth and discoloration of teeth in the fetus [5]. While minocycline has shown a higher rate of treatment resolution, oral azithromycin has also demonstrated successful outcomes in several reports [3]. Azithromycin works by blocking bacterial ribosomal translation via interaction with the 23S rRNA located on the 50S subunit of ribosomes. Reported side effects include QT prolongation, gastrointestinal upset, and hepatotoxicity [6]. While azithromycin has an overall good safety profile in pregnancy, risks of possible congenital malformations in the first trimester have been reported in cohort studies [7].

While its chemical structure differs from macrolide antibiotics, clindamycin shares a similar mechanism of action with azithromycin by also binding to the 23S rRNA ribosomal 50S subunit of bacteria [8]. Because the patient presented in the first trimester, topical clindamycin was prescribed due to its low risk of systemic absorption and compatibility with pregnancy [9]. Clindamycin 1% lotion was selected to target the proposed pathophysiology of abnormal host reaction to bacteria in CARP, while glycolic acid was recommended to mitigate abnormal keratinocyte proliferation.

## Conclusions

Topical clindamycin phosphate 1% lotion along with glycolic acid 5% gel-cream appears to represent an effective therapeutic approach for patients diagnosed with CARP. Although minocycline has historically offered the greatest success rate, the use of topical clindamycin serves as a unique alternative for patients who may be pregnant or apprehensive about systemic therapies and their possible side effects. Further studies are needed to support the effectiveness of topical clindamycin usage in individuals diagnosed with CARP.
